# Internal jugular vein duplication

**DOI:** 10.4103/0970-0358.59303

**Published:** 2009

**Authors:** Paolo Biondi, Giuseppe Colella, Giulio Gherardini, Gianpaolo Tartaro, Raffaele Rauso

**Affiliations:** Maxillo-Facial Surgery, Head and Neck Department, II University of Naples, Naples, Italy; 1 Plastic Surgery, Rome, Italy

Sir,

The internal jugular vein is the largest vein in the neck and drains the intracranial structures and deep structures of the face and neck. It runs the length of the neck slightly lateral to the common carotid artery within the carotid sheath and, on the right, crosses in front of the right subclavian artery, to join the subclavian vein.[1] Duplication of the internal jugular vein is a rare finding.

We came across a case of duplication of right internal jugular vein and offer some clinical comments on the importance of this rare anatomical feature.

**Figure 1:**
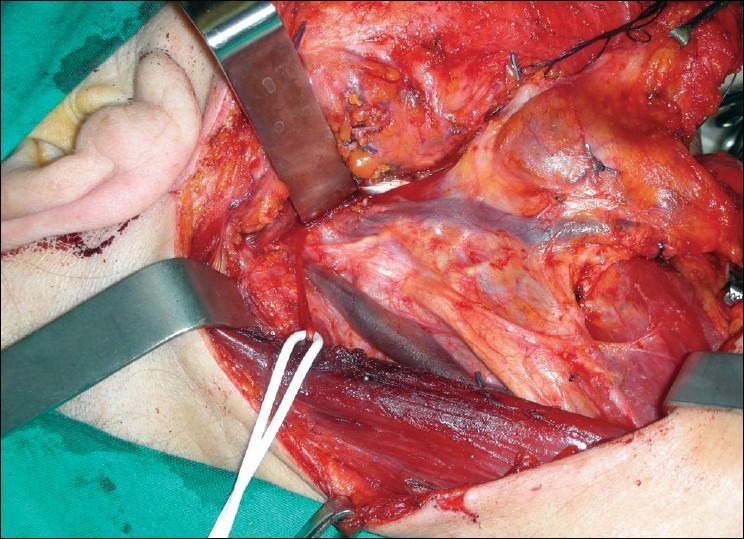
The bifurcation of the right internal jugular vein discovered during neck dissection

A 65-year-old woman with a T2N0M0 squamous cell carcinoma of the right margin of the tongue had a local wide excision and an ipsilateral modified type 3 radical neck dissection. During the dissection of the neck a bifurcation of the right internal jugular vein, about 2cm from the jugular foramen was encountered [Figure 1]. Both branches of the internal jugular vein had the same thickness and poured into the right subclavian vein. The anterior branch was parallel to the carotid artery and received the common facial vein, the superior and inferior thyroid veins and the transverse cervical vein. The posterior branch passed within the carotid sheath, drained only the cerebral blood and emptied into the subclavian vein lateral to the medial branch. None showed evidence of phlebectasia or aneurysm. The spinal accessory nerve passed between the medial and lateral branches, exactly superficial to the medial branch and under the lateral branch.

Duplication of the internal jugular vein is a rare congenital anomaly. The vein divides into two branches that separately enter the subclavian vein. Almost always it involves the upper third of the vein.[2,3] In our case, the duplication was 2 cm below the base of the skull.

Duplication of the internal jugular vein is usually reported in association with phlebectasia, which is a soft non-pulsatile cervical swelling that increases in size during Valsalva maneuver.[4] In our case, no aneurysm or phlebectasia was observed.

Three theories have been formulated to explain duplication:[3] the vascular theory, that is usually accepted[3] the neural hypothesis and the bony hypothesis. Duplication is thought to result from the appearance of a secondary venous ring at a lower level surrounding the spinal accessory nerve during foetal life.[4] The persistence of this secondary ring in adult life may be important in the aetiology of venous duplication.[4]

Unexpected duplication of the internal jugular vein could impact specific clinical procedures, creating the possibility of either iatrogenic morbidity or incorrect diagnosis.

In our case, the large area involved and additional sites for ligature complicated selective neck dissection.
